# Nasopharyngeal Carcinoma with Cystic Cervical Metastasis Masquerading as Branchial Cleft Cyst: A Potential Pitfall in Diagnosis and Management

**Published:** 2017-03

**Authors:** Lum Sai-Guan, Kong Min-Han, Ngan Kah-Wai, Mohd-Razif Mohamad-Yunus

**Affiliations:** 1 *Department of Otorhinolaryngology – Head and Neck Surgery, University Kebangsaan Malaysia Kebangsaan Medical Centre (UKMMC), Kuala Lumpur, Malaysia*.; 2 *Department of Pathology, Hospital Serdang, Selangor, Malaysia.*

**Keywords:** Branchial cleft cyst, Metastasis, Nasopharyngeal carcinoma

## Abstract

**Introduction:**

Most metastatic lymph nodes from head and neck malignancy are solid. Cystic nodes are found in 33% - 61% of carcinomas arise from Waldeyer’s ring, of which only 1.8% - 8% originate are from the nasopharynx. Some cystic cervical metastases were initially presumed to be branchial cleft cyst. This case report aims to highlight the unusual presentation of cystic cervical metastasis secondary to nasopharyngeal carcinoma in a young adult. The histopathology, radiological features and management strategy were discussed.

**Case Report:**

A 36-year-old man presented with a solitary cystic cervical swelling, initially diagnosed as branchial cleft cyst. Fine needle aspiration yielded 18 ml of straw-coloured fluid. During cytological examination no atypical cells were observed. Computed tomography of the neck showed a heterogeneous mass with multiseptation medial to the sternocleidomastoid muscle. Histopathological examination of the mass, post excision, revealed a metastatic lymph node. A suspicious mucosal lesion at the nasopharynx was detected after repeated thorough head and neck examinations and the biopsy result confirmed undifferentiated nasopharyngeal carcinoma.

**Conclusion:**

Cystic cervical metastasis may occur in young patients under 40 years. The primary tumour may not be obvious during initial presentation because it mimicks benign branchial cleft cyst clinically. Retrospective review of the computed tomography images revealed features that were not characteristic of simple branchial cleft cyst. The inadequacy of assessment and interpretation had lead to the error in diagnosis and subsequent management. Metastatic head and neck lesion must be considered in a young adult with a cystic neck mass.

## Introduction

Neck swelling due to cervical lymph node metastasis is the most common presenting symptom of patients with nasopharyngeal carcinoma. Most of the cervical metastases are solid. Cystic swelling is detected in 33% - 61% of cases (1,2). The incidence of unsuspected carcinoma in cervical cysts, initially presumed to be of branchial cleft origin, has been reported in 11% - 21% of cases (3). The majority of cystic cervical metastases originate from oropharyngeal carcinoma especially palatine tonsils, whereas nasopharyngeal carcinoma accounts only for 1.8% - 8% (2,4). Most of the malignant cystic cervical swellings were reported in adult patients aged over 40 years but rarely in younger patient. We present a young adult with cystic cervical metastasis secondary to nasopharyngeal carcinoma who was initially diagnosed as branchial cleft cyst, and review the management strategy for this unusual presentation. 

## Case Report

**Fig 1:**
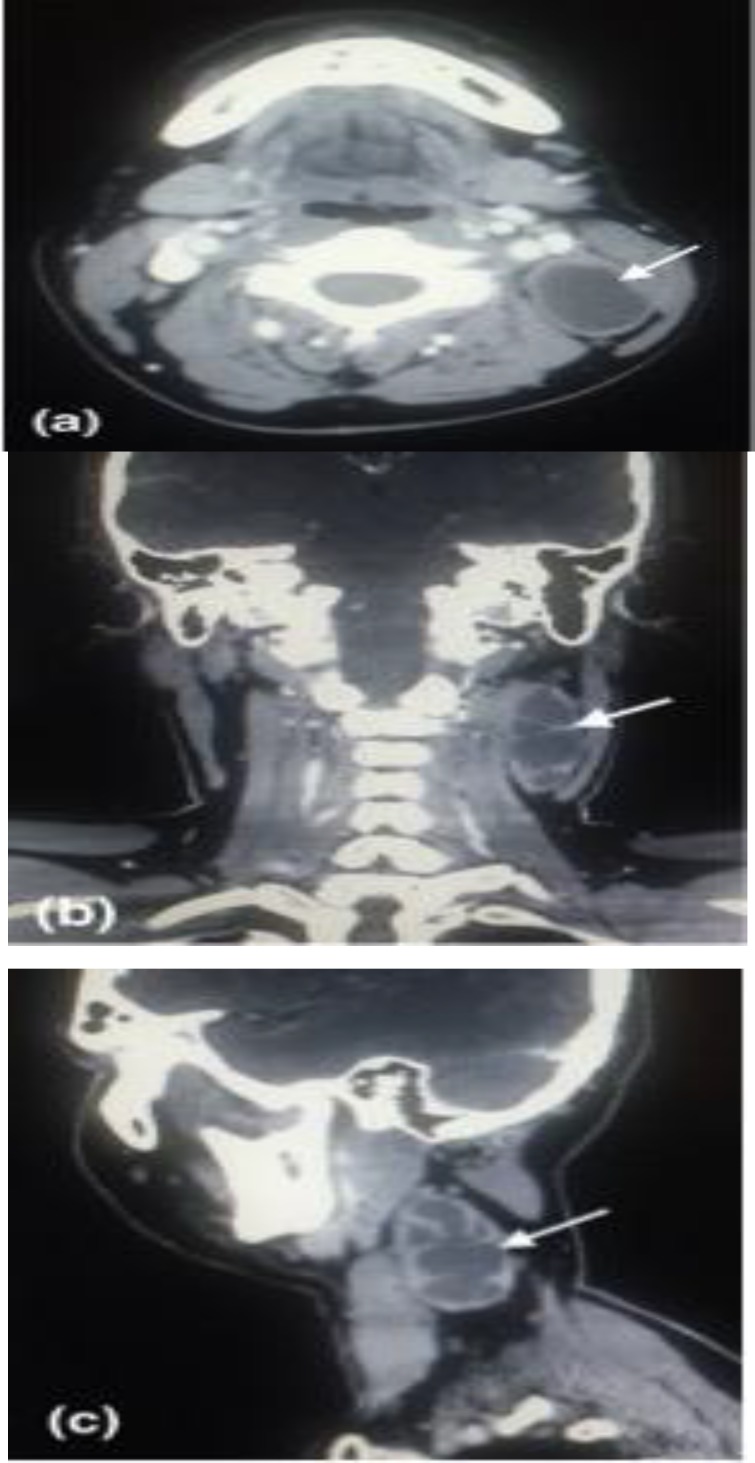
**(a)** Axial CT scan shows a cystic mass (white arrow) deep to left sternocleidomastoid muscle.  **(b)** & **(c) **Coronal and sagittal reconstruction CT images reveal multiple septa within the cystic mass

A 36-year-old man presented with three weeks history of progressively enlarging left neck swelling. Clinical examination revealed a cystic mass at the level II of left neck anterior to sternocleidomastoid muscle, measured 5 cm x 5 cm. It was soft, fluctuant, mobile, mildly tender on palpation with normal overlying skin. Nasopharyngeal endoscopy did not detect any abnormality then. The total white blood cells count was raised at 12,000/mm (3). He was treated as infected left branchial cleft cyst with oral amoxicillin/ clavulanate. However, he returned three weeks later with increasing pain over the cystic neck swelling. Fine needle aspiration (FNA) yielded 18 ml of straw-colored fluid. Cytological examination of the fluid did not show any atypical cell. Computed tomography (CT) of the neck showed a solitary heterogeneous mass medial to sternocleido- mastoid muscle, measured 5.6 cm x 3.1 cm x 2.4 cm, with multiple septa within (Fig. 1). 

**Fig 2:**
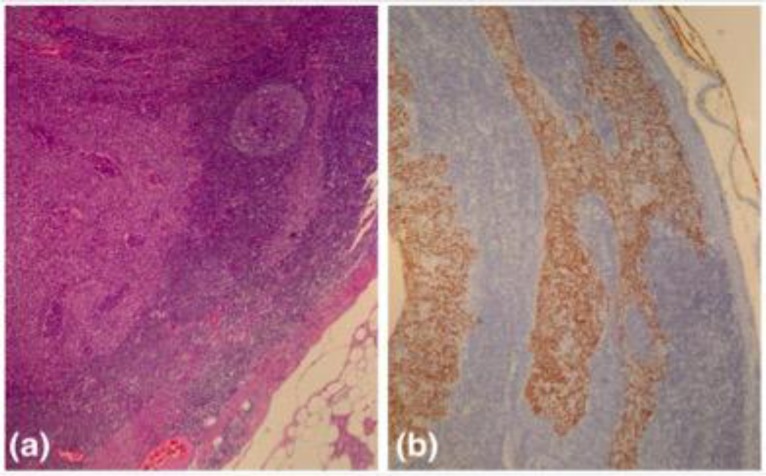
(a) Cyst wall lined by sheets of malignant cells in a lymphoid rich stroma (H & E stain, x40). (b) The tumor cells highlighted by epithelial marker, AE1/AE3 (Immunohistochemical stain, x40

The diagnosis was not revised and the patient underwent excision of the left neck cystic mass later under general anesthesia. The cyst was easily removed and intraoperatively no tract was found connecting the cyst to pharynx or hyoid bone. Histological examination of the cystic mass revealed a lymph node with cystic degeneration, showing cohesive sheets of malignant cells in lymphocyte and plasma-cell-rich stroma (Fig.2). 

The tumour cells demonstrate large vesicular nuclei and prominent nucleoli. There was no definite squamous or glandular differentiation seen. Immunohistochemical studies showed that tumour cells were highlighted with cytokeratin stain (AE1/AE3) but negative for CD3, CD20, CD30, CK7, CK20, TTF-1, HMB45 and Ki67. These findings support the diagnosis of metastatic undifferentiated carcinoma with possible primary tumor at head and neck region. The patient was thus reinvestigated thoroughly and repeated nasopharyngeal endoscopy revealed a suspicious mucosal lesion at the left fossa of Rosenmuller. Biopsy of the lesion was taken and histopathology report subsequently confirmed non-keratinizing undifferentiated nasopharyngeal carcinoma. The patient eventually received radiotherapy to the nasopharynx and cervical region five months after the first presentation. There was no evidence of recurrence at 12-months follow-up. 

## Discussion

Patients presenting with a cystic cervical mass may constitute a diagnostic challenge to the otorhinolaryngologist, as it can be benign or malignant in nature. The commonest cause in patients below 30 year-old is branchial cleft cyst. Most of the literatures cited that malignant cervical cystic swelling are more common in over 40s age group, of which 80% are secondary lymph node metastasis from head and neck carcinoma (5). As a result of preconception that carcinomas of head and neck are diseases of the middle-aged and elderly, little attention has been paid to the development of carcinomas in the young (6).

The patient in this case presented at the age of 36 with solitary left neck cystic swelling, misguiding the otorhinolaryngologist to diagnose it as branchial cleft cyst at first consultation, without suspicion of malignancy as differential diagnosis. Several authors have concluded that a solitary cystic cervical mass in patients above 40 years should be presumed to be carcinoma until proven otherwise (1,5). It is now evident that secondary cystic metastasis from head and neck malignant tumor should always be considered even though patient was below 40 year-old, especially in South-East Asian region where incidence of nasopharyngeal carcinoma is high. 

Tsukuda et al. reported that 4.3% of head and neck carcinomas occurred in patients aged less than 40 years, most frequently from nasopharynx (60.4%), and histopathology analysis characteristically revealed undifferentiated carcinoma (6). In addition, they found that metastatic cervical lymph nodes secondary to nasopharyngeal carcinoma were significantly higher in patients younger than 40 years (93%) than older age group (67%).

In the past, cases of cystic cervical metastasis with occult primary have been labeled as branchiogenic carcinoma. This is referred to malignant tumors arising from the vestigial remnants of the branchial pouches. However, most authors now question its existence as a true clinicopathologic entity (7). There has been increasing evidence of association between a solitary cystic cervical metastasis and an occult primary tumour in the tonsils, base of tongue or nasopharynx (3,8). Other less common primary sites are the thyroid, lower lip, oral cavity, hypopharynx and larynx. The younger patient population often present with cystic metastatic swelling than solid metastatic carcinoma (3).The cystic metastasis appears to have a better prognosis compared to other metastatic squamous cell carcinomas of the head and neck, with 5-year survival rate of 77% and 10-year survival rate of 50% (4).

In view of possibility of malignancy in an adult with cervical cystic mass, efforts should be made to look for primary tumour especially in Waldeyer’s ring. The assessment of a patient who presents with a cystic cervical mass should include a thorough head and neck examination, in particular tonsils, base of tongue and endoscopic examination of nasopharynx. Any suspicious mass should be biopsied for histopathology examination. FNA is a simple investigation and has been shown to be helpful in solid cervical mass to achieve correct diagnosis. However, the sensitivity was low in the diagnosis of both malignant cystic metastases and branchial cysts (73% and 60% respectively) (8). Some authors have cited high false negative rate (50% - 67%) of FNA in the diagnosis of malignancy in cystic metastases due to low cell density of the aspirate for cytological evaluation (1,2). The false negative result may leads to false reassurance thus resulting in delay in searching for the primary tumor site, in addition to delay in the initiation of the appropriate oncologic treatment. Radiological studies such as ultrasonography and CT scan may aid in the diagnosis of a cervical cystic mass. Features that are in favour of branchial cleft cyst are well circumscribed oval-shaped lesion, with a thin wall that is non-contrast enhanced, with a hypodense and homogeneous content, and without septations. Conversely, an irregular lesion with a marked peripheral contrast enhancement, invasion of adjacent structures, internal vascularization, heterogeneous content and septa suggest a nodal metastasis (4). The size of the lesion does not correlate with the nature of the tumour (9). 

The treatment of cystic cervical metastasis differs from that of benign branchial cyst. Therefore, accurate diagnosis of a cystic cervical swelling is of paramount importance. In case the primary is occult, many authors have suggested performing panendoscopy with bilateral tonsillectomy and random biopsies of fossa of Rosenmuller and base of tongue (3,4). Emmanuel et al*.* reported that undifferentiated nasopharyngeal carcinoma (39%) was the most common diagnosis after random biopsy during panendoscopy (4) Some authors objected excision of the neck mass to avoid inadvertent excision of a possible cystic lymph node metastasis (5). The excision of cervical cyst also causes violation of the neck before definitive treatment, which may increase risk of local recurrence and wound complications. Furthermore, the management protocol for nasopharyngeal carcinoma is distinct from other head and neck carcinoma. It is radiosensitive and first line treatment would consist of chemoradiation to both the primary site and the cervical metastasis. Surgical excision is reserved for persistent or recurrent neck disease. 

## Conclusions

Cystic cervical metastasis may occur in a young patient with nasopharyngeal carcinoma. It may mimic features of branchial cleft cyst clinically, leading to incorrect diagnosis. This resulted in delay of treatment and thus compromise the patient’s outcome. There should be high suspicion of malignancy in an adult presenting with a cystic cervical mass, even if the primary site is not obvious. Nasopharyngeal examination including random biopsy is highly recommended before excision of the cystic neck mass. Thorough head and neck examinations, accurate interpretation and correlation of histopathology and radiological imaging results are all essential to achieve the correct diagnosis and management. 
